# Access to HIV Services at Non-Governmental and Community-Based Organizations among Men Who Have Sex with Men (MSM) in Cameroon: An Integrated Biological and Behavioral Surveillance Analysis

**DOI:** 10.1371/journal.pone.0122881

**Published:** 2015-04-23

**Authors:** Claire E. Holland, Erin Papworth, Serge C. Billong, Sethson Kassegne, Fanny Petitbon, Valentin Mondoleba, Laure Vartan Moukam, Isaac Macauley, Simon Pierre Eyene Ntsama, Yves Roger Yomb, Jules Eloundou, Franz Mananga, Ubald Tamoufe, Stefan D. Baral

**Affiliations:** 1 Johns Hopkins Bloomberg School of Public Health, Department of Epidemiology, Baltimore, Maryland, 21205, United States of America; 2 Central Technical Group, National AIDS Control Committee, Ministry of Public Health, Yaoundé, Cameroon; 3 Population Services International, Cotonou, Benin; 4 CARE International in Cameroon, Yaoundé, Cameroon; 5 Cameroon National Association for Family Welfare (CAMNAFAW), Yaoundé, Cameroon; 6 Association Camerounaise pour le Marketing Sociale (ACMS), Yaoundé, Cameroon; 7 Alternatives-Cameroun, Douala, Cameroon; 8 Humanity First, Yaoundé, Cameroon; 9 Global Viral Cameroon, Yaoundé, Cameroon; David Geffen School of Medicine at UCLA, UNITED STATES

## Abstract

**Background:**

Men who have sex with men (MSM) are more likely to be living with HIV than other adult men in low- and middle-income countries. MSM experience barriers to accessing HIV services including a lack of available specialized care, and community-level stigma and discrimination. This study aims to examine the uptake of HIV services at non-governmental and community-based organizations (NGOs/CBOs) to identify ways to improve coverage of HIV prevention and treatment among MSM.

**Methods:**

An Integrated Biological and Behavioral Surveillance (IBBS) survey was conducted in Yaoundé and Douala, Cameroon in 2011 using the respondent driven sampling (RDS) method to recruit and interview 239 MSM in Yaoundé and 272 MSM in Douala.

**Results:**

MSM in Yaoundé were statistically significantly more likely to have accessed NGO/CBO services or been reached by an outreach worker in the past 12 months if they had any STI symptoms (aOR 2.17 CI 1.02-4.59. p=0.04), or if they had a larger MSM social network (aOR 1.02 CI 1.01-1.04. p<0.01). MSM in Douala were more likely to have accessed NGO/CBO services or been reached by an outreach worker in the past 12 months if they were living with HIV (aOR 3.60 CI 1.35-9.60. p=0.01), or if they reported higher numbers of male sexual partners (aOR 1.17 CI 1.00-1.36. p=0.046). Compared to men in Douala, MSM in Yaoundé were significantly less likely to have accessed NGO/CBO services or been reached by an outreach worker in the past 12 months (aOR 0.22 CI 0 .14-0.34. p=<0.01).

**Conclusions:**

With appropriate funding and resources, community-based organizations that provide care specifically for MSM can improve access to HIV prevention, treatment, and care services. Additionally, using social networks to reach MSM can connect greater numbers of the population to effective HIV interventions, which will improve health outcomes and decrease onward transmission of HIV.

## Key Messages

Men who have sex with men carry a disproportionately high burden of HIV in Cameroon, and they experience individual, community-level, and structural risks of HIV infection as well as significant barriers to accessing HIV prevention and treatment services.

More established community-based organizations that provide care specifically for men who have sex with men can improve access to health services among the population.

Scaling up community-led HIV interventions and leveraging social networks of men who have sex with men can decrease barriers to care and increase health service uptake.

## Introduction

Men who have sex with men (MSM) have a much higher burden of HIV than other adult men in low and middle-income countries, including countries with a traditionally generalized HIV epidemic (>1.0% prevalence) [1–3]. The Central African nation of Cameroon is one example with an HIV prevalence among the population of reproductive age (ages 15–49) of 4.5% in 2012 [4] and HIV prevalence of 37% among MSM in Yaoundé and Douala in 2011 [5]. Several factors contribute to an increased risk of HIV transmission and acquisition among MSM, including a greater biological risk of HIV transmission during anal intercourse, versatile sexual positioning that facilitates rapid HIV transmission within sexual networks, and limited access to prevention and care services due to community-level stigma, discrimination and criminalization of same sex practices [6–9].

MSM often have difficulties accessing HIV prevention and care services due to fear of discrimination or arrest, and denial of service provision because of their sexuality [10–13]. These factors, among others, have been demonstrated to reduce accessibility and uptake of HIV prevention, treatment, and care services among MSM across countries of Sub-Saharan Africa (SSA) [8,11,14]. In addition, given that there are many countries that criminalize same-sex practices in SSA, data have consistently demonstrated limited provider awareness about the specific health needs of MSM and significant levels of health-care related stigma [15–17]. Considering the burden of HIV, the significant unmet needs of HIV prevention and treatment services, and the barriers to health service delivery and uptake, MSM represent a key population in the HIV response across the continent [1,7,8,18].

Early engagement into the continuum of HIV care is essential for preventing clinical progression to AIDS and decreasing onward transmission of HIV [19]. The continuum of HIV care is a range of HIV services beginning with diagnosing unknown HIV infections and ending with viral suppression achieved through retention in antiretroviral therapy (ART) [19]. Individual viral suppression as a result of HIV treatment has been proven to decrease HIV transmission in a population through high coverage of ART among all individuals living with HIV in a community. The impact of this ART coverage and viral suppression is often referred to as lowering the community viral load [20]. Retention at each step of the continuum of HIV care is challenging even among the general population [21,22], and although evidence-based HIV interventions have been identified for MSM at the individual, network, and structural level [10,15,23,24], MSM must continuously access these interventions for them to be effective.

Strategies for improving engagement and retention in the continuum of HIV care have been evaluated in many contexts to determine effective ways of increasing access to and uptake of HIV services [25–27]. To help mitigate barriers to care and improve access to HIV services for key populations in diverse settings, various models of HIV prevention, treatment, and care services have been proposed: full integration into general population services, hybrid models, and stand-alone clinics [28]. Across studies, decentralized HIV care [29] and community-based interventions have been shown to improve health care access among MSM populations. In Brazil, the influence of a community-based social network facilitated the sharing of health-related information [30] and encouraged attendance at health care services [31]. Constructive relations with health service providers and the use of peer educators have also been shown to connect MSM to HIV prevention and care [32]. Furthermore, health workers with specific training on the structural, community, and individual barriers that MSM face have been able to effectively provide specialized care to the population and overcome certain barriers to care [23,33,34]. Although large public hospitals often provide subsidies and are therefore more financially accessible, they commonly lack specialized MSM services which can discourage care seeking, especially for MSM sexual health needs [31]. Conversely, the Avahan program in India, a large-scale behavioral intervention designed specifically to deliver care to MSM, demonstrated increased care seeking behavior in the country [35]. This evidence illustrates that appropriately-funded non-governmental organizations (NGOs) and community-based organizations (CBOs) may improve access to HIV prevention and care interventions among MSM by providing MSM specific services and decreasing stigma [31,36].

Although NGO and CBO services can improve care seeking among MSM, low levels of engagement in HIV prevention and treatment services persist in many contexts. Identifying socio-demographic or sexual history characteristics that facilitate or prevent MSM from accessing NGO/CBO services may allow NGOs and CBOs to better target this population and engage hard-to-reach subgroups within the MSM population. Furthermore, comparing access to NGO/CBO care in different cities provides information on regional variation of NGO/CBO services and can identify the most effective methods of engaging MSM in the continuum of HIV care. This study, conducted in collaboration with the National AIDS Control Committee (NACC) and Ministry of Health in the two largest cities in Cameroon, aims to contribute knowledge that can improve availability and access to NGOs and CBOs for HIV prevention and treatment services among MSM by examining current trends and associations of care seeking in the population.

## Methods

### Study population

The data for this study were collected through an Integrated Biological and Behavioral Surveillance (IBBS) cross-sectional survey conducted in Yaoundé and Douala from August to September 2011 and completed with support from the NACC and the Ministry of Health. Eligibility requirements for participation included being born a man, being at least 18 years of age, and reporting at least one sexual act, whether anal or oral, with another man in the 12 months preceding the survey.

### Sampling method

The survey was administered using the respondent-driven sampling (RDS) method [37]. The IBBS sampling design used seven seeds with diverse sexual identities and sexual role preferences as a baseline in each city. One local NGO and two CBOs, which provide services specifically to MSM and Lesbian, Gay, Bisexual, Transgender, and Intersex (LGBTI) communities, were involved in the recruitment of seed respondents [5]. The minimum sample size per site was calculated to be 241 men based on predicting a 15% change in the prevalence of condom use at last anal intercourse from a baseline of 60%, with a design effect of 2.0, a precision level of. 05 and a power of 80%. The final sample consisted of 239 MSM in Yaoundé and 272 in Douala [5].

### Study procedure

Each eligible respondent answered structured survey questions regarding subjects including: their socio-demographics; network size; sexual behaviors; and access to CBO-run MSM centers (which included outreach services) [5]. The administration of the questionnaire lasted approximately 40 minutes, following which, study participants agreed to be tested for HIV and syphilis. Individuals who agreed (90.4%; 462/511) were sent for pre-test counseling with a trained counselor and tested using a ~4ml blood specimen collected by a phlebotomist. Within a short time span (30 minutes to an hour) test results were provided by the same counselor during a post-test counseling session. Participants who screened positive were provided referrals for appropriate health care [5].

### Ethical review

Written informed consent was provided by all participants and ethical approval was granted by the Cameroon National Ethics Committee in 2011. Secondary data analysis of the behavioral and HIV seroprevalence data was approved by the Institutional Review Board of the Johns Hopkins Bloomberg School of Public Health in 2012.

### Laboratory testing

HIV and syphilis testing was conducted under the Ministry of Public Health in Cameroon guidelines. Tests included Determine HIV-1/2 (Inverness Medical, Chiba, Japan) and Human HEXAGON HIV 1+2 (Human GmBh, Wiesbaden, Germany). Confirmatory testing was completed on all indeterminate and positive samples as well as 15% of the negative samples using HIV enzyme-lined immunosorbent assay (ELISA). Syphilis testing was conducted using Rapid Protein Reagin (RPR; SGM Italia, Rome, Italy) and Treponema pallidum hemagglutination assay (TPHA; Fortress Diagnostics Limited, Anrtim, UK). Testing and quality control was conducted by a local research laboratory, Global Viral Forecasting in Yaoundé, Cameroon. A full description of serological testing and results is provided in Park et al 2013; S1 File [5].

### Statistical analysis

Analysis of NGO/CBO access and MSM HIV prevalence was conducted separately among participants from Douala and Yaoundé to determine the reach and impact of these services. All results in this analysis are reported in crude and RDS-adjusted weights and were conducted in Stata 12 [38]. RDS adjustment was calculated using the RDSII estimator based on the differences in the social network sizes of participants [5]. Homophily was assessed on a scale of -1 to +1 to determine how likely respondents are to recruit other individuals like themselves [37]. Access to NGO/CBO services was measured as having visited the NGO or CBO service center in the past 6 months or being reached by a CBO peer educator in the past 12 months. RDS weighted prevalence and bootstrapped confidence intervals were calculated for variables included in the adjusted regression and displayed in Table 1.

**Table 1:** Characteristics of MSM recruited from Douala (N = 272) and Yaoundé (N = 239), 2011.

	All	Douala		Yaoundé	
	n (%)	n (%)	Respondent Driven Sampling weighted % (95%CI)	n (%)	Respondent Driven Sampling weighted % (95%CI)
Total	511 (100)	272 (100)	-	239 (100)	-
Age, median (IQR) (yrs)	24 (21–28)	23 (21–27)	-	25 (21–28)	-
18–23	238 (46.6)	142 (52.2)	57.5 (50.4–64.6)	96 (40.2)	42.2 (34.1–50.3)
24–29	185 (36.2)	85 (31.3)	29.7 (23.3–36.1)	100 (41.8)	42.4 (43.3–50.4)
30+	88 (17.2)	45 (16.5)	12.8 (8.2–17.4)	43 (18.0)	15.4 (9.1–21.8)
NGO/CBO service access in the past 12 months	302 (59.1)	202 (74.3)	66.1 (57.5–74.7)	100 (41.8)	33.4 (26.3–40.6)
HIV status: HIV+	171 (37.0)	73 (28.6)	25.7 (19.2–32.1)	98 (47.3)	44.0 (35.2–52.8)
Previous history of HIV testing	413 (81.6)	216 (80.6)	77.5 (71.0–84.0)	197 (82.8)	79.7 (73.5–86.0)
Education					
Primary or less	26 (5.1)	20 (7.4)	7.6 (3.9–11.3)	6 (2.5)	2.7 (.3–7.0)
Secondary	341 (66.7)	183 (67.3)	70.1 (63.9–76.4)	158 (66.1)	70.1 (63.0–76.9)
Higher than secondary	144 (28.2)	69 (25.4)	22.3 (16.6–28.0)	75 (31.4)	27.3 (20.7–33.9)
Occupational status					
Student or Apprentice	204 (39.9)	116 (42.6)	46.6 (40.0–53.2)	88 (36.8)	36.9 (29.4–44.5)
Employed	248 (48.5)	126 (46.3)	44.4 (37.8–50.9)	122 (51.0)	48.7 (40.6–56.8)
Unemployed	59 (11.6)	30 (11.0)	9.1 (5.4–12.7)	29 (12.1)	14.4 (7.9–20.8)
MSM Network size, median (IQR)	12 (6–25)	13 (5–25)	-	12 (6–25)	-
Number of male partners in last 12 months	3 (2–5)	3 (2–5)	-	3 (2–5)	-
Sexual identity					
Gay/homosexual	144 (28.2)	71 (26.1)	23.3 (17.3–29.2)	73 (30.5)	27.8 (20.5–35.0)
Had male and female sexual partners in past 12 months	114 (22.3)	49 (18.0)	20.0 (14.2–25.8)	65 (27.2)	28.5 (21.1–36.0)
Relationship status:					
Single	425 (84.2)	226 (84.6)	85.6 (80.5–90.6)	194 (83.3)	87.3 (82.2–92.4)
In a relationship or married	80 (15.8)	41 (15.4)	14.4 (9.4–19.5)	39 (16.7)	12.7 (7.6–17.8)
Inconsistent condom use with casual partners in the past 12 months	207 (40.5)	112 (41.2)	38.8 (31.8–45.9)	96 (40.2)	38.4 (31.3–45.6)
STI symptoms in the past 12 months	175 (34.6)	80 (29.9)	31.0 (23.9–38.2)	95 (39.9)	39.2 (31.8–46.5)

Data on factors associated with HIV infection and prior HIV testing are available in S1 and S2 Files.

Non RDS weighted and RDS weighted logistic regression of NGO/CBO access was run to determine statistically significant (p<0.05) associations of MSM accessing NGO/CBO services. Age was a fixed covariate in the adjusted model that did not confound the predictive value of other covariates in multiple logistic regression. The independent variables were selected based on previous analyses conducted with the data, prior knowledge, and literature [5]. Backward and forward stepwise selection were used to identify variables that should be highly considered in the final model. HIV testing was excluded from the final regression model because testing was viewed as a result of accessing services. The final multiple logistic regression model included age, HIV status, education, occupational status, sexual identity, history of STI symptoms, size of MSM social network, having male and female sexual partners in the past 12 months, number of male sexual partners, and condom use. Collinearity was checked on all models using the vif command and on covariates in a pairwise correlation coefficient test. The final model had mean vif of 1.85 in Yaoundé and 1.66 in Douala. The model was checked for fit using a Hosmer-Lemeshow goodness of fit test and the resulting p-value was greater than. 05 in both cities. Additionally Akaike information criterion (AIC) was used and the final model had the lowest AIC in Yaoundé and the second lowest AIC in Douala.

Multiple logistic regression of NGO/CBO access was also conducted on the total sample to determine statistically significant (p<0.05) differences of accessing NGO/CBO services between the cities. This logistic regression was non RDS weighted as each city has a different RDS network. The adjusted regression includes the same variables that were used in the final model for each individual city.

## Results

In Yaoundé, 247 MSM were screened and 239 met the eligibility criteria, consented and participated in the study. There were 296 MSM screened in Douala and 272 who met the eligibility criteria, consented and participated in the study. In Yaoundé the median number of waves per seed was 5 (range 1–9) and in Douala the median number of waves per seed was 6 (range 1–8) [5]. Homophily in the group that had never accessed NGO/CBO services was-.12 in Yaoundé and. 09 in Douala and. 16 and. 29 in the group that had accessed services in those cities respectively. Homophily was close to zero in both groups and the assumptions of the RDS network for NGO/CBO access were satisfied by random recruitment. Results are reported as RDS weighted proportions followed by crude percentages in parentheses.

### Participant characteristics and health service access

The majority of the participant sample was between the ages of 18–23, 42.2% (40.2; 96/239) in Yaoundé and 57.5% (52.2; 142/272) in Douala. In Yaoundé 44.0% (47.3; 98/207) of MSM were living with HIV and in Douala 25.7% (28.6; 73/255) of MSM were living with HIV. The MSM network size of participants was similar in both cities with a mean of 12 in Yaoundé and a mean of 13 in Douala, and the number of male partners in the last 12 months was 3 in each city. The majority of the sample in both cities had previously been tested for HIV with 79.7% (82.8; 197/238) in Yaoundé and 77.5% (80.6; 216/268) in Douala. The outcome of interest in this study of having accessed NGO/CBO service at a health center or being reach by a peer educator found that 33.4% (41.8; 100/239) of MSM in Yaoundé and 66.1% (74.3; 202/272) of MSM in Douala had been connected to NGO/CBO services in the past 12 months.

### Correlates of NGO/CBO access in Yaoundé and Douala

In Yaoundé, two variables showed a significant relationship with NGO/CBO access. MSM who experienced STI symptoms were 2.19 (CI 1.16–4.12. p<0.05) times more likely to have accessed NGO/CBO services in the non-weighted multivariable regression and 2.17 (CI 1.02–4.59. p<0.05) times more likely in the RDS-weighted regression. MSM network size was also significantly associated with accessing NGO/CBO services in Yaoundé. The odds of accessing NGO/CBO services increased for each additional MSM in a respondent’s network with an odds ratio 1.02 (CI 1.00–1.03. p<0.05) in the non-weighted model and 1.02 (CI 1.01–1.04. p<0.01) in the RDS weighted model (Table 2).

**Table 2:** Bivariate and multivariate models of the correlates of access to NGO/CBO services and outreach among MSM in Yaoundé (N = 239), 2011.

	NGO/CBO access (n = 100)	No NGO/CBO access (n = 139)	OR (95%CI)	RDS-weighted OR (95%CI)	aOR (95%CI)	p-value	RDS-weighted aOR (95%CI)	p-value
Age								
Per year increase for MSM aged 18–23	33 (33.0)	63 (45.3)	Ref	Ref	Ref	.849*	Ref	.814*
Per year increase for MSM aged 24–29	44 (44.0)	56 (40.3)	1.50 (0.84–2.67)	1.26 (0.65–2.42)	1.23 (0.60–2.52)	.570	1.18 (0.53–2.64)	.683
Per year increase for MSM aged 30+	23 (23.0)	20 (14.4)	2.20 (1.06–4.57)	1.91 (0.83–4.43)	1.19 (0.44–3.22)	.737	1.46 (0.45–4.75)	.526
HIV positive	43 (43.0)	55 (39.6)	1.15 (0.66–2.01)	0.97 (0.52–1.82)	1.02 (0.54–1.91)	.952	0.77 (0.37–1.60)	.484
History of HIV testing	88 (88.0)	109 (78.4)	1.95 (0.94–4.04)	1.50 (0.65–3.44)	-	-	-	-
Education: Higher than secondary	38 (38.0)	37 (26.6))	1.69 (0.97–2.93)	2.00 (1.07–3.76)	1.59 (0.81–3.15)	.174	1.68 (0.80–3.54)	.172
Occupational status								
Student or Apprentice	33 (33.0)	55 (39.6)	Ref	Ref	Ref	.193*	Ref	.380*
Employed	57 (57.0)	65 (46.8)	1.46 (0.84–2.56)	1.29 (0.68–2.44)	1.59 (0.76–3.33)	.218	1.52 (0.66–3.51)	.326
Unemployed	10 (10.0)	19 (13.7)	0.88 (0.36–2.11)	0.83 (0.30–2.27)	0.75 (0.28–2.02)	.562	0.76 (0.27–2.16)	.611
Self identities as gay	34 (34.0)	39 (28.1)	1.32 (0.76–2.30)	1.34 (0.71–2.53)	0.99 (0.51–1.91)	.973	0.96 (0.43–2.17)	.931
Any STI symptoms in past 12 months	48 (48.0)	47 (33.8)	1.79 (1.06–3.03)	1.98 (1.09–3.61)	**2.19 (1.16–4.12)**	**.015**	**2.17 (1.02–4.59)**	**.043**
Per increase in MSM social network	-	-	1.01 (1.01–1.03)	1.03 (1.01–1.05)	**1.02 (1.00–1.03)**	**.038**	**1.02 (1.01–1.04)**	**.009**
Had male and female sexual partners in past 12 months	24 (24.0)	41 (29.5)	0.75 (0.42–1.36)	0.83 (0.42–1.64)	0.67 (0.33–1.34)	.259	0.76 (0.33–1.77)	.525
Per increase in number of male sexual partners	-	-	1.03 (0.98–1.09)	1.05 (1.00–1.10)	1.02 (0.95–1.08)	.619	1.03 (0.96–1.10)	.443
Inconsistent condom use with casual partner(s)	42 (42.0)	54 (38.8)	0.82 (0.58–1.16)	1.17 (0.61–2.25)	0.64 (0.31–1.31)	.222	0.69 (0.30–1.60)	.382

Abbreviations: RDS, respondent driven sampling; OR, odds ratio; aOR, adjusted odds ratio; CI, confidence interval.

Adjusted odds ratio model includes all variables listed in Table 2 except history of HIV testing.

*Overall Wald test p-value for categorical variables calculated using testparm.

In Douala, HIV status was significantly associated with access to NGO/CBO services in both non-weighted and RDS-weighted regression. MSM who were living with HIV had significantly higher odds of having accessed NGO/CBO services than MSM who were not living with HIV with an odds ratio of 3.46 (CI 1.44–8.34. p<0.01) in the non-weighted multivariable regression and 3.60 (CI 1.35–9.60. p<0.05) in the RDS-weighted regression. Age was significantly associated with accessing NGO/CBO services in the non-weighted regression however, after correcting for bias in the respondent driven sampling method, age was no longer significantly associated with NGO/CBO service access. The odds ratio for each increase in male sexual partners was 1.11 (CI 1.00–1.23. p = 0.054) in the non-weighted model and 1.17 (CI 1.00–1.36. p<0.05) in the RDS-weighted model. Men with a greater number of male sexual partners had significantly higher odds of having accessed NGO/CBO services than men with fewer male sexual partners, though significance was only achieved after adjusting for the RDS network (Table 3). Additionally, crude analysis conducted on the total sample adjusting for all variables in the final regression model showed that MSM in Yaoundé were statistically significantly less likely to have accessed NGO services than MSM in Douala with an odds ratio of 0.22 (CI 0.14–0.34. p<0.01).

**Table 3:** Bivariate and multivariate models of the correlates of access to NGO/CBO services and outreach among MSM in Douala (N = 272), 2011.

	NGO/CBO access (n = 202)	No NGO/CBO access (n = 70)	OR (95%CI)	RDS-weighted OR (95%CI)	aOR (95%CI)	p-value	RDS-weighted aOR (95%CI)	p-value
Age								
Per year increase for MSM aged 18–23	105 (52.0)	37 (52.9)	Ref	Ref	Ref	.021*	Ref	.425*
Per year increase for MSM aged 24–29	57 (28.2)	28 (40.0)	0.72 (0.40–1.29)	0.94 (0.48–1.82)	**0.42 (0.21–0.87)**	**.020**	0.65 (0.29–1.49)	.314
Per year increase for MSM aged 30+	40 (19.8)	5 (7.10)	2.81 (1.03–7.68)	2.66 (0.84–8.37)	1.43 (0.45–4.48)	.543	1.36 (0.37–4.97)	.640
HIV positive	65 (32.2)	8 (11.4)	3.71 (1.67–8.23)	4.40 (1.77–10.9)	**3.46 (1.44–8.34)**	**.006**	**3.60 (1.35–9.60)**	**.011**
History of HIV testing	172 (85.1)	44 (62.9)	3.62 (1.91–6.84)	3.73 (1.82–7.65)	-	-	-	-
Education: Higher than secondary	52 (25.7)	17 (24.3)	1.08 (0.58–2.03)	1.13 (0.55–2.31)	1.33 (0.62–2.82)	.465	1.22 (0.53–2.82)	.629
Occupational status								
Student or Apprentice	85 (42.1)	31 (44.3)	Ref	Ref	Ref	.482*	Ref	.243*
Employed	97 (48.0)	29 (41.4)	1.22 (0.68–2.19)	1.31 (0.68–2.54)	1.00 (0.99–1.02)	.992	0.97 (0.45–2.11)	.942
Unemployed	20 (9.90)	10 (14.3)	0.73 (.31–1.73)	0.49 (0.19–1.28)	0.52 (0.17–1.61)	.259	0.38 (0.11–1.25)	.111
Self identities as gay	55 (27.2)	16 (22.9)	1.26 (0.67–2.39)	1.50 (0.72–3.13)	0.76 (0.35–1.63)	.485	0.69 (0.27–1.77)	.436
Any STI symptoms in past 12 months	61 (30.2)	19 (27.1)	1.16 (0.63–2.14)	0.82 (0.41–1.61)	0.98 (0.49–1.95)	.959	0.69 (0.31–1.50)	.346
Per increase in MSM social network	-	-	1.02 (1.00–1.03)	1.02 (1.00–1.04)	1.00 (0.99–1.02)	.315	1.01 (0.99–1.04)	.318
Had male and female sexual partners in past 12 months	32 (15.8)	17 (24.3)	0.59 (0.30–1.14)	0.47 (0.23–0.99)	0.64 (0.28–1.46)	.286	0.48 (0.19–1.25)	.134
Per increase in number of male sexual partners	-	-	1.08 (1.01–1.16)	1.10 (1.00–1.21)	1.11 (1.00–1.23)	.054	**1.17 (1.00–1.36)**	**.046**
Inconsistent condom use with casual partner(s)	67 (33.2)	32 (45.7)	0.68 (0.38–1.19)	0.55 (0.27–1.11)	0.71 (0.34–1.46)	.351	0.73 (0.32–1.66)	.448

Abbreviations: RDS, respondent driven sampling; OR, odds ratio; aOR, adjusted odds ratio; CI, confidence interval.

Adjusted odds ratio model includes all variables listed in Table 3 except history of HIV testing.

*Overall Wald test p-value for categorical variables calculated using testparm.

## Discussion

### Health service access among MSM in Cameroon

This study examined levels of NGO/CBO access among MSM in Yaoundé and Douala and identified characteristics associated with accessing HIV care. The data show that there was a higher level of service uptake among MSM in Douala. Controlling for the effects of age, HIV status, and other social and sexual characteristics MSM in Douala were more likely to have accessed NGO/CBO services than MSM in Yaoundé. Although MSM in Douala were more connected to NGO/CBO health services, a study conducted in Douala in 2012 found that only 58% of MSM in the sample population had been exposed to HIV prevention interventions [39] indicating that there remain significant proportions of the MSM community that are not yet reached by NGO/CBO health services.

The socio-demographic and sexual history characteristics associated with having accessed NGO/CBO care were different in each location demonstrating that a diverse subgroup of MSM are connected to NGO and CBO services in Yaoundé and Douala. In Yaoundé a larger MSM social network was associated with access to NGO/CBO services demonstrating the potential impact of network-based interventions, and the importance of reaching MSM with smaller social networks that are difficult to engage in care. The results also show that MSM who were living with HIV in Douala were more likely to have accessed NGO/CBO services while MSM living with HIV in Yaoundé were not more likely to have accessed NGO/CBO services than MSM who are not HIV infected. This identifies a possible gap in NGO/CBO HIV services among MSM living with HIV in Yaoundé. However, HIV testing in Yaoundé did not differ by NGO/CBO service access, so some MSM in Yaoundé likely access HIV prevention services in different health centers. The differences in NGO/CBO service access in these cities may be influenced by the social context for MSM in Yaoundé and Douala and the existing health services in each city.

### Models of service delivery for MSM

As the two largest cities in Cameroon, Douala and Yaoundé each have at least one MSM HIV health service organization, but these organizations differ in size and scope. The CBO in Douala, Alternatives-Cameroun, is the longest running CBO serving LGBTI populations in the country [40]. Humanity First in Yaoundé was established in the same year the study took place, and conducted primarily peer outreach in the community, not offering many services onsite. The Cameroon National Association for Family Welfare (CAMNAFAW), the NGO health center in Yaoundé, is not a MSM specific organization but recently expanded HIV services targeted for MSM. These health centers provide an example of two different models of health service delivery. Alternatives-Cameroun in Douala is an example of a stand-alone service, while CAMNAFAW in Yaoundé integrates MSM-supportive services into existing general population services. These models of care have achieved significantly different levels of service access in each city highlighting the importance of evaluating the effectiveness of service provision to determine if the model of care is reaching the MSM population. The success of regional service models relies on identifying the appropriate model for each context and evaluating service targets along the continuum of HIV care.

### Community-based interventions and the continuum of HIV care

Humanity First and Alternatives-Cameroun are community-led organizations, and capitalizing on local MSM and LGBTI community leadership can help inform service model implementation throughout Cameroon [41]. Involving local leaders in the development of interventions can help increase service access and connection to the MSM community [14,41]. In Yaoundé there was a statistically significant relationship between accessing NGO/CBO services and a larger MSM social network. MSM community leaders can help link existing MSM social networks to prevention and care services, which encourages care seeking among individuals within the social network. This has also been shown in China where a large MSM network was associated with improved HIV knowledge and participation in HIV prevention [42]. Additionally, organizations specifically dedicated to LGBTI health have been shown to facilitate community-level support systems [41,43]. These support networks and social interactions can establish and spread care-seeking behavior within the MSM population [31]. Locally developed and regionally specific services, integrated within existing social networks and community structure, may help bridge the gap to HIV services for the MSM population and improve retention in the continuum of HIV care.

Community leadership is also needed to provide specialized services for MSM considering several structural factors that contribute to difficulties in care seeking. Cameroon is one of more than seventy countries around the world that criminalize same-sex conduct [44], and one that prosecutes individuals for same-sex practices [45,46]. Community-level stigma and discrimination can further create a challenging context for care seeking and service delivery and has been reported as a key deterrent to care seeking among MSM in the country [47–50]. Developing NGO/CBO services with regional MSM community leaders may help identify the most appropriate model of service delivery and improve access to care, given the complexity of this context.

The uptake of services at NGOs and CBOs in Cameroon and in similar studies regarding MSM access to health care, suggest that these organizations can provide entry into the continuum of care through tailored peer outreach, prevention and supportive services for LGBTI communities [31–33,35]. Capacity to initiate and retain MSM in the continuum of care is needed in all of the service delivery models as mentioned above. Furthermore since clinical treatment is an essential element of the continuum that facilitates a reduction of HIV transmission [19], NGOs and CBOs have a potential role in providing specific MSM-focused clinical care in complex environments.

### Limitations

A limitation of this study is that it was only conducted in two cities in Cameroon. To further examine regional differences in NGO/CBO services it would be beneficial to conduct research that includes diverse study sites around the country. Another limitation is that data was not collected on what proportion of HIV diagnoses were previously unknown diagnoses. Future analyses, including an assessment of HIV health services for MSM in Cameroon and an evaluation of MSM engagement at each step in the continuum of HIV care starting with undiagnosed HIV, could inform health service access. The sampling method may have produced bias toward men who have accessed NGO/CBO services as the organizations that collaborated in the study attracted participants that were more likely to have accessed their services, however RDS adjustment was used to correct for this bias in the sampling method, and the levels of NGO/CBO service access in the RDS adjustment are lower than the crude proportions. The sample is also skewed toward younger MSM with the majority in the 18–23 year old group. The younger MSM may have also increased the bias toward NGO/CBO access since a statistically significantly higher proportion of 18–23 year olds reported accessing NGO/CBOs in the crude sample. Additionally, RDS has been used successfully in multiple Sub-Saharan African countries to access similar hidden populations, and the weighted results have increased representativeness of the MSM target population [3].

## Conclusions

These analyses revealed significant regional variation in NGO/CBO service access and in HIV prevalence among MSM in Cameroon. The regional differences in care seeking support a decentralized, community-based approach to HIV service provision that relies on leveraging existing MSM social networks to increase the uptake of health services. Community leadership should be able to better address specific barriers to HIV care and treatment for MSM supporting the provision of non-discriminatory, contextually appropriate, and accessible HIV services. Taken together, the data presented here provide empiric data highlighting the importance of community in optimizing the continuum of HIV care and treatment for MSM especially in stigmatizing environments. Consistent data indicate that primary HIV prevention and high ART coverage result in a lower community viral load and decrease new infections, which will help combat HIV among MSM on a national level. The high burden of HIV together with clear and unmet needs among these men necessitate improved coverage of supportive and comprehensive HIV prevention, treatment and care services for all men who have sex with men in Cameroon.

## Supporting Information

S1 File HIV prevalence and factors associated with HIV infection among men who have sex with men in Cameroon [5]. (PDF)

S2 File Correlates of prior HIV testing among men who have sex with men in Cameroon: a cross-sectional analysis [51]. (PDF)
