# Supplementary material for: Access to HIV Services at Non-Governmental and Community-Based Organizations among Men Who Have Sex with Men (MSM) in Cameroon: An Integrated Biological and Behavioral Surveillance Analysis
Source: PLoS One. 2015 Apr 23;10(4):e0122881. doi: 10.1371/journal.pone.0122881 (PMC4408025; doi:10.1371/journal.pone.0122881)
Supplement: S2 File — (PDF) [file pone.0122881.s002.pdf]

Park et al. BMC Public Health 2014, 14:1220
http://www.biomedcentral.com/1471-2458/14/1220

RESEARCH ARTICLE Open Access

Correlates of prior HIV testing among men who have sex with men in Cameroon: a cross-sectional analysis

Ju Nyeong Park[1*], Erin Papworth[1], Serge Clotaire Billong[2], Jean Bosco Elat[2], Sethson Kassegne[3], Ashley Grosso[1], Laure Moukam[4], Isaac Macauley[5], Yves Roger Yomb[6], Valentin Mondoleba[2], Jules Eloundou[7], Matthew LeBreton[8], Sosthenes Charles Ketende[1] and Stefan Baral[1]

Abstract

Background: Regular HIV testing is vital for timely linkage to the HIV care continuum and ensuring the success of behavioral and biomedical interventions to prevent HIV acquisition. Men who have sex with men (MSM) are a key population for HIV prevention, treatment, and care efforts globally. This study measures the factors associated with prior HIV testing among MSM in Cameroon.

Methods: In 2011, 272 and 239 MSM aged ≥ 18 were recruited from Douala and Yaoundé respectively using respondent-driven sampling (RDS) for a cross-sectional surveillance study. Participants completed a structured socio-behavioral survey and were offered HIV and syphilis testing and counseling.

Results: The majority of men self-reported ever testing for HIV (81.6%; 413/506) and receiving their last HIV test result (95.4%; 394/413). Testing in the last 12 months was more prevalent in Douala (63.3%; 169/267) compared to Yaoundé (55.9%; 132/236). Median frequency of testing was every 18 months in Douala and every two years in Yaoundé. In multivariate RDS-weighted analysis, correlates of ever testing for HIV in Douala were: having higher than secondary education compared to having secondary education or less (aOR = 3.51, 95% CI: 1.32-9.34), ever accessing a community-based HIV service for MSM (aOR = 3.37, 95% CI: 1.57-7.24) and having ≥4 male oral or anal sexual partners in the past 12 months (aOR = 2.49, 1.08-5.74). In Yaoundé, having higher than secondary education (aOR = 7.96, 95% CI: 1.31-48.41) was associated with ever testing for HIV.

Conclusions: Supporting regular HIV testing and linkage to care is important in a context of high HIV prevalence and limited use of condoms and condom-compatible lubricants. Building the capacity of MSM organizations and mainstream health services to deliver affordable, integrated, confidential, and MSM-sensitive HIV testing may assist in effectively engaging more MSM in the HIV treatment cascade. Giving specific attention to MSM who are younger, of lower socioeconomic status and less connected to community-based MSM organizations may increase HIV testing uptake. Given the levels of HIV testing and high HIV prevalence among MSM in Cameroon, optimizing the safe and effective provision and uptake of antiretroviral-based prevention and treatment approaches is paramount in changing the trajectory of the HIV epidemic among these men and within their sexual networks.

Keywords: Men who have sex with men (MSM), HIV/AIDS, HIV testing, Voluntary counseling and testing (VCT), Epidemiology, Africa, Respondent-driven sampling (RDS), Homosexuality, Prevention, Risk factors, Health behaviors

* Correspondence: ju.park@jhu.edu
[1]Center for Public Health and Human Rights, Department of Epidemiology, Johns Hopkins Bloomberg School of Public Health, Baltimore, USA
Full list of author information is available at the end of the article

© 2014 Park et al.; licensee BioMed Central Ltd. This is an Open Access article distributed under the terms of the Creative Commons Attribution License (http://creativecommons.org/licenses/by/4.0), which permits unrestricted use, distribution, and reproduction in any medium, provided the original work is properly credited. The Creative Commons Public Domain Dedication waiver (http://creativecommons.org/publicdomain/zero/1.0/) applies to the data made available in this article, unless otherwise stated.

Background

Globally, men who have sex with men (MSM) are disproportionately affected by HIV compared to other men of reproductive age [1-3]. HIV-related epidemiologic studies among this key population in the Central and West African region are relatively sparse but consistently demonstrate high HIV prevalence [4]. HIV prevalence of 25.5% in Douala and 44.4% in Yaoundé, Cameroon among MSM were observed in the nation's first HIV surveillance study for MSM in 2011 [5].

Regular HIV testing is critical for timely HIV diagnosis, and is the first step in engaging people living with HIV in the HIV treatment cascade. Informing individuals of their serostatus may also promote changes in HIV preventive behaviors, such as increasing the frequency of condom use during penile-anal or vaginal intercourse [6]. HIV testing venues are an important gateway for the delivery of HIV prevention and treatment services including behavioral approaches, such as individualized risk reduction counseling, and biomedical approaches, such as universal coverage of antiretroviral therapy (ART) for those living with HIV, and the provision of pre-exposure prophylaxis (PrEP) for those at high risk of HIV acquisition.

While additional studies are warranted, the percentage of MSM who have ever been tested for HIV significantly varies across sub-Saharan Africa, and within countries, with ever testing prevalences of 35% to over 90% in Southern Africa [7-12], 11% and 88% in Senegal [13,14], 17% and 55% in Nigeria [15,16], and 81% in Douala, Cameroon [17]. In comparison, an estimated 90% of MSM recruited from 21 United States cities reported prior HIV testing, with approximately 62% tested in the previous 12 months [18]. An estimated 50% of MSM in the U.S. who tested positive for HIV were newly diagnosed [18].

Documented individual-level factors positively associated with ever receiving an HIV test among MSM in the sub-Saharan African setting include employment [15], bisexual concurrency [19], and openly identifying as gay or homosexual as compared to straight or bisexual [20]. In one study in Douala, Cameroon, knowing someone living with HIV, being exposed to HIV prevention programs, having a steady male partner, and being non-Muslim was associated with ever testing [17]. HIV testing in the past 12 months has been shown to be associated with using condoms during the last male-to-male sexual encounter in Lesotho [12].

Among South African MSM, never testing for HIV was associated with being black, living in a township and lack of HIV-related knowledge [21]. Moreover, lower income and internalized homophobia reduced the likelihood of recent HIV testing [20,21]. Self-reported reasons for never testing for HIV in South Africa included low risk perception and perceived health care stigma [8]. Fear of public exposure of sexual practices and identity have also been reported as significant barriers for MSM seeking HIV testing in qualitative studies conducted in Senegal and Kenya [22]. Fear of testing was associated with preferring a feminine gender expression, being sexually active, having a history of sexually transmitted infections (STI), and experiencing sexual orientation-based victimization at school and the workplace [8]. Finally, studies from sub-Saharan Africa have demonstrated that heteronormative HIV prevention messaging may increase misperceptions that penile-anal sex does not pose a risk for HIV transmission and deter MSM from being tested [10,23].

Sexual relationships between men are both criminalized and highly stigmatized in Cameroon, similar to many countries across the world, posing a policy-level challenge when delivering HIV-related services to MSM [24,25]. Perceived or experienced stigma and discrimination due to sexual orientation and concerns about confidentiality in the healthcare setting are structural barriers to accessing HIV services, particularly in settings where male-to-male sexual practices are criminalized [8,26-30]. The invisibility of MSM in health surveillance programs and limited funding for targeted, affordable, confidential and MSM-sensitive HIV testing and treatment services in Cameroon may also pose policy-level barriers to the delivery of HIV-related services in a setting of high HIV prevalence [5,27,31-35].

In the current analysis, we aim to contribute to the limited literature on HIV testing among MSM in Central and West Africa by characterizing HIV testing practices among MSM in Douala and Yaoundé and investigating the correlates of ever receiving an HIV test.

Methods

Study population

This cross-sectional study was conducted between August-September 2011 in Douala and Yaoundé, the two largest cities of Cameroon. The details of the study have been described elsewhere [5]. Briefly, men aged 18 years or older who reported engaging in penile-anal or oral intercourse with another man in the last 12 months were eligible for the study. Participants were recruited in each city using respondent-driven sampling (RDS), a sampling technique that minimizes biases that may arise from peer-based recruitment [36]. Seven seeds heterogeneous in sexual orientation and sexual role preference were selected through existing community contacts and community-based organizations (CBOs) serving MSM to begin the recruitment process in each city.

Consent was provided orally and documented in writing by the interviewer. Study procedures were anonymous. The study was approved by the Cameroon National Ethics Committee, and secondary data analysis was approved by

the Johns Hopkins Bloomberg School of Public Health Institutional Review Board.

Data collection

Participants completed an interviewer-administered structured survey containing questions on socio-demographics, network size, sexual behaviors including condom and lubricant use (always versus often, sometimes or never), experiences of STI symptoms, access to community-based MSM centers (which included outreach services), access to free condoms, and HIV counseling and testing (HCT) experiences. Participants were also asked 13 true/false items on knowledge of HIV transmission routes, prevention and treatment options, and eight social support items on whether social contacts "encourage you to consistently use condoms". Social contact types included partners, family and peers (response options strongly agree/agree/disagree/strongly disagree).

All participants were compensated 1000 CFA Franc (2 USD) for their time and transport costs at the initial study visit and an additional 1000 CFA Franc (2 USD) for every peer referred into the study (maximum of three). All participants also received free HIV and syphilis testing, pre- and post-test counseling, condoms, condom-compatible lubricants (CCL), and access to peer education and support groups. Newly diagnosed individuals were referred to HIV care and treatment.

Statistical analysis

Non-seed participants who answered the question on ever testing for HIV ("Have you ever been tested for HIV" (yes/no)) were included in the current analysis. Unweighted prevalence, RDS-weighted prevalence and bootstrapped confidence intervals were calculated for HIV testing variables in descriptive analysis. Individualized weights were created in Stata/SE Version 11.2 (College Station, Texas) using the RDS-II estimator to account for differences in social network size for each variable included in the descriptive analysis [37]. Network size was assessed using the response from the latter of two questions: "How many men who have had oral or anal sex with men in the last 12 months do you know, who also know you and live in this city?" and "among these men that you know personally, how many of them are 18 years and older?".

Differences in willingness to return to a HCT site by testing venue type were assessed using the Pearson Chi-square test. Fisher's exact test was used to assess differences in ever testing and HIV testing in the past 12 months ("How many times have you been tested for HIV during the last 12 months" (≥1/None)) by current HIV serostatus and age category (coded as 18-21/22-25/26-29/ ≥30 years) for each city.

Condom use variables were dichotomized (consistent use coded as 0 from the response "always use condoms in the past 12 months"; inconsistent use coded as 1 from "most of the time/sometimes/never use condoms in the past 12 months"). The HIV knowledge composite variable was constructed by taking the sum of correct answers (possible range 0–13) and converting it to a percentage (possible range 0-100%). The social support for condom use composite variable was created by dichotomizing each of the 8 items into yes (strongly agree and agree) and no (disagree and strongly disagree), then taking the sum of the 8 items (possible range 0–8), and converting it into a percentage (possible range 0-100%).

Separately for each city, unweighted bivariate logistic regression models were used to estimate the unadjusted association between ever HIV testing and covariates, which were selected based on the published literature. Bivariate logistic regression models using RDS-weighting were also built. Weights for all regression models were built using the RDS-II estimator using "ever tested for HIV" as the outcome. Homophily (range: −1 to +1) was assessed to evaluate whether individuals preferred to recruit MSM with the same HIV testing outcome.

Unweighted multivariate logistic regression models were built to estimate the adjusted association between ever HIV testing and covariates, with spline terms for age (knot at age of 30 years) forced into all models regardless of statistical significance. The Akaike information criterion (AIC) was used to favor the most parsimonious model. P values <0.05 were used to indicate statistical significance. RDS-weighted multivariate logistic regression models, using the same weights as used in bivariate models, were also built. RDS-naïve and RDS-adjusted multivariate regression models were also built using the same methodology with HIV testing in the past 12 months as the outcome. Complete case analysis was used for all regression modeling as missingness was <2% for all variables. A sensitivity analysis was conducted to test the effect of clustering by seed in the final multivariate models. All data analyses and weighting were conducted in Stata/SE Version 11.2 (College Station, Texas).

Results

Two hundred and seventy-two and 239 men from Douala and Yaoundé respectively were recruited into the study. Overall, the median age was 24 years (range 18–51, interquartile range (IQR) 21–28). In both cities, the majority had completed secondary education (66.7%; 341/511), and identified their relationship status as single (84.2%; 425/505). Sixty-two percent of men chose "bisexual" (425/511) to describe their sexual orientation, whereas 28.6% chose "gay or homosexual" (144/511), 8.0% chose "MSM" (41/511) and 1.8% chose "other" (9/511). In Douala, median number of waves per seed was six (range 1–8). In Yaoundé, median number of waves per seed was five (range 1–9). A detailed description of the demographic

and sampling characteristics has been published previously [5]. The current analysis was restricted to participants who completed questions on HIV testing (n = 268 in Douala; n = 238 in Yaoundé).

HIV testing practices

HIV testing prevalence and practices are summarized in Figure 1 and Table 1. Overall, 81.6% (413/506) of MSM had ever been tested for HIV. In Douala and Yaoundé, RDS-adjusted prevalence of ever HIV testing were 77.5% and 79.9%, and HIV testing in the past 12 months were 63.2% and 54.3% respectively. Homophily of ever testing was 0.15 among men in Douala and 0.21 among men in Yaoundé. The median frequency of testing since being sexually active with a man was once every 18 months in Douala and once every two years in Yaoundé (Table 1).

Both ever tested for HIV (p = 0.001) and HIV testing in the past 12 months (p = 0.004) differed in Douala by HIV serostatus: 6.9% of men who tested HIV-positive in this study had never been tested for HIV prior to the study (5/72), compared to 24.6% among men who tested HIV-negative (44/179). Similarly, 22.2% of men who tested HIV-positive had not been tested in the past 12 months (16/72) compared to 42.1% among men who tested HIV-negative (75/178). In Yaoundé, no differences by HIV serostatus was observed: 17.4% (17/98) of men who tested HIV-positive had never been tested compared to 20.4% (22/108) of HIV-negative men (p = 0.60), and 53.6% (52/97) of men who tested HIV-positive had not been tested in the past 12 months compared to 50.9% (55/108) of HIV-negative men (p = 0.78).

Ever tested for HIV and tested in the past 12 months did not differ by age category in Douala (p = 0.14 and p = 0.28 respectively). Ever tested for HIV did differ by age category in Yaoundé (p = 0.001) however no age difference was observed for testing in the past 12 months (p = 0.07) (Figure 1).

In both cities, the majority were advised to get tested by peer educators (74%; 301/407), and rarely by healthcare providers (1.7%; 7/407). Of men who had ever been tested, 95.9% (394/413) received their last test result. The most common reason for testing in Douala and Yaoundé was to know one's status (81.5% and 65.8%) followed by being required for a medical procedure at a hospital (8.1% and 14.8%) (Table 1).

Testing venue patterns differed between the cities (Table 1); in Douala, Alternatives-Cameroun, a CBO specifically serving the MSM community of the city, was the most common place last tested (45.2%; 95% CI: 35.9-54.4%) followed by public hospitals (31.1%; 95% CI: 22.4-39.8%). This was in contrast to Yaoundé where public hospitals (47.9%; 95% CI: 38.8-57.1%), followed by private hospitals (25.2%; 95% CI: 17.0-33.4%) and other services were more accessed than the local CBO-based service targeting MSM. Homophily was 0.29 for men who had accessed Alternatives-Cameroun (Douala) in the past 12 months compared to 0.09 for men who had not; homophily was 0.11 for men who had accessed Humanity First (Yaoundé) in the past 12 months compared to −0.17 for men who had not. Information on HIV testing in both cities was commonly received through mass media campaigns, CBO-based HIV services and friends; and only 6.5% (33/506) of participants received information on HIV testing from a doctor or nurse.

Willingness to return to a HCT site

The majority of men who were last tested at a CBO-based HCT site were willing to return to the same site in both Douala and Yaoundé (98.9% vs. 100.0%). Willingness to return to other HCT sites such as a public or private hospital was comparatively lower (Table 2).

Factors associated with prior HIV testing: Douala

The statistical significance of variables did not change between the RDS-naïve and RDS-weighted bivariate analyses. The unadjusted RDS-weighted odds of ever tested for HIV increased per year rise between age 18 and 29 (OR: 1.12 per year, 95% CI: 1.00-1.26) then declined

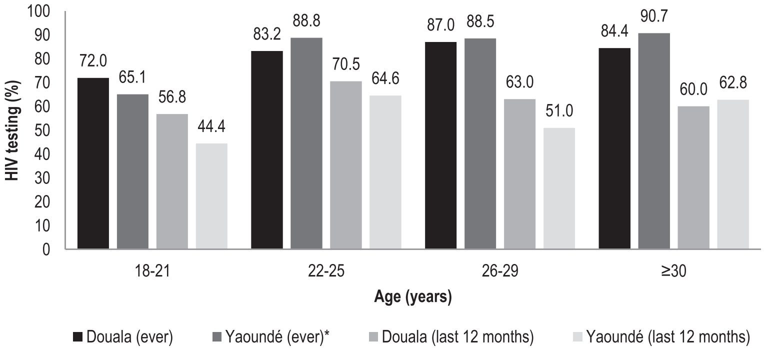

Figure 1 Prior HIV testing among men who have sex with men recruited in 2011 from Douala (n = 268) and Yaoundé (n = 238) stratified by age group.

Table 1 HIV testing practices of men who have sex with men (MSM) recruited from Douala (n = 268) and Yaoundé (n = 238), Cameroon 2011

	All	Douala		Yaoundé	
	n (%)	n (%)	RDS- weighted % (95% CI)	n (%)	RDS- weighted % (95% CI)
Total	506 (100)	268 (100)	-	238 (100)	-
Never tested for HIV	93 (18.4)	52 (19.4)	22.5 (15.7-29.1)	41 (17.2)	20.1 (13.8-26.5)
Intend to get tested in next 12 months	88 (95.7)	49 (94.2)	-	39 (95.1)	-
Ever tested for HIV	413 (81.6)	216 (80.6)	77.5 (70.8-84.3)	197 (82.8)	79.9 (73.5-86.2)
No. of times tested per two years of being sexually active with other men, median (IQR)	1.2 (0.5-2.3)	1.3 (0.6-3)	-	1 (0.5-2)	-
Received test results at last test	394 (95.4)	204 (94.9)	94.3 (89.5-99.1)	190 (96.9)	97.6 (95.1-1.00)
Disclosed test result to someone	314 (79.7)	148 (72.6)	73.8 (65.9-81.7)	166 (87.4)	86.0 (80.5-91.5)
Tested in last 12 months	301 (59.8)	169 (63.3)	63.2 (55.0-71.3)	132 (55.9)	54.3 (46.0-62.6)
Place last tested^					
Public hospital or clinic	162 (40.2)	66 (32.0)	31.1 (22.4-39.8)	96 (48.7)	47.9 (38.8-57.1)
Private hospital or clinic	71 (17.6)	30 (14.6)	13.1 (7.6-18.6)	41 (20.8)	25.2 (17.0-33.4)
CBO HIV service for MSM	110 (27.3)	89 (43.2)	45.2 (35.9-54.4)	21 (10.7)	6.8 (2.8-10.7)
Mobile unit or van, university, event, other	60 (14.9)	21 (10.2)	10.6 (5.9-15.3)	39 (19.8)	20.1 (12.1-28.0)
Advised to get tested by^#:					
Peer educator	301 (74.0)	172 (81.5)	-	129 (65.8)	-
Sexual partner	46 (11.3)	17 (8.1)	-	29 (14.8)	-
No-one (myself)	21 (5.2)	7 (3.3)	-	14 (7.1)	-
Doctor, nurse, other health professional	7 (1.7)	4 (1.9)	-	3 (1.5)	-
Other	32 (7.9)	11 (5.2)	-	21 (10.7)	-
Information on HIV testing ever received from^:					
Radio, television, posters	142 (27.8)	58 (21.3)	26.3 (16.3-36.3)	84 (35.1)	34.5 (26.1-42.8)
CBO HIV service for MSM	102 (20.0)	50 (18.4)	26.5 (18.0-35.0)	52 (21.8)	21.3 (15.7-26.8)
Friend	131 (25.6)	73 (26.8)	36.8 (26.0-47.6)	58 (24.3)	23.7 (17.8-29.6)
Family	46 (9.0)	9 (3.3)	4.3 (1.1-7.5)	37 (15.5)	14.4 (9.5-19.3)
Doctor or nurse	33 (6.5)	19 (7.0)	6.1 (2.3-10.0)	14 (5.9)	4.8 (2.0-7.5)
None of these sources	2 (0.5)	2 (0.7)	-	0 (0.0)	-

^Among ever tested.
#RDS-weighted estimates could not be run due to small cell sizes.
CBO, community-based organization; CI, confidence interval; HIV, Human Immunodeficiency Virus; IQR, interquartile range; MSM, men who have sex with men; RDS, respondent-driven sampling.

Table 2 Willingness to return to a voluntary HIV counseling and testing (HCT) site by testing venue type among men who have sex with men (MSM) in Douala (n = 200) and Yaoundé (n = 174), Cameroon 2011

	Community-based HIV service targeting MSM n (%)	Public hospital n (%)	Private hospital n (%)	Other n (%)	p-value
Douala					
Would return to this HCT site	87 (98.9)	52 (82.5)	23 (79.3)	15 (75.0)	**0.03**^
Yaoundé					
Would return to this HCT site	18 (100.0)	79 (90.8)	29 (82.9)	29 (85.3)	0.54

^RDS-weighted Pearson's chi-square test.

every year from age 30 (OR: 0.78 per year, 95% CI: 0.64-0.95) (Table 3). Other bivariate associations with ever tested for HIV were having higher than secondary education compared to lower than or equal to secondary education (OR: 3.96, 95% CI: 1.62-9.67), non-Christian religion (OR: 0.17, 95% CI: 0.38-0.78), ever accessing CBO service for MSM including outreach services (OR: 3.69, 95% CI: 1.90-7.58) and having four or more male oral or anal sexual partners in the past 12 months (OR: 2.88, 95% CI: 1.31-6.31).

In RDS-naïve multivariate logistic analysis, factors independently associated with ever tested for HIV were age (aOR: 1.12 per year increase for ages 18–29, 95% CI: 1.00-1.25; aOR: 0.80 per year increase for ages ≥30, 95% CI: 0.64-0.98), and ever accessing a community-based HIV service for MSM (aOR: 3.23, 95% CI: 1.63-6.38).

Table 3 Bivariate and multivariate models of the factors associated with HIV testing among MSM in Douala (n = 268), Cameroon 2011

	Ever tested for HIV n (%)	Never tested for HIV n (%)	RDS-weighted OR (95% CI)	RDS-weighted aOR (95% CI)	p-value
Total	216 (80.6)	52 (19.4)	-	-	-
Age (years)					
18-29 (per year increase in age)	178 (79.8)	45 (20.2)	**1.12 (1.00-1.26)**	1.08 (0.95-1.21)	0.2
≥30 (per year increase in age)	38 (84.4)	7 (15.6)	**0.78 (0.64-0.95)**	0.84 (0.68-1.05)	0.1
Highest level of education attained					
≤Secondary	154 (77.4)	45 (22.6)	Ref	Ref	-
University or technical studies	62 (89.9)	7 (10.1)	**3.96 (1.62-9.67)**	**3.51 (1.32-9.34)**	**0.01**
Occupational status					
Student or apprentice	92 (80.0)	23 (20.0)	Ref	-	-
Employed	99 (79.8)	25 (20.16)	0.87 (0.42-1.78)	-	-
Unemployed	25 (86.2)	4 (13.8)	2.29 (0.69-7.67)	-	-
Christian religion[1]	181 (78.4)	50 (21.7)	**0.17 (0.38-0.78)**	-	-
Sexual orientation: Gay[2]	55 (78.6)	15 (21.4)	0.50 (0.24-1.08)	-	-
Sexual role preference: Receptive[3]	73 (85.9)	12 (14.1)	1.60 (0.71-3.59)	1.38 (0.60-3.21)	0.5
Relationship status: Single[4]	179 (79.2)	47 (20.8)	0.55 (0.15-2.04)	-	-
Ever accessed CBO HIV service for MSM	172 (86.4)	27 (13.6)	**3.69 (1.90-7.58)**	**3.37 (1.57-7.24)**	**0.002**
Ever received free condoms	162 (82.7)	34 (76.5)	1.59 (0.74-3.41)	-	-
In the last 12 months:					
Any STI symptom	62 (77.5)	18 (22.5)	0.82 (0.39-1.71)	-	-
Had male and female sexual partners[5]	66 (82.5)	14 (17.5)	1.47 (0.74-2.95)	-	-
Number of male partners					
1-3	129 (76.3)	40 (23.7)	Ref	Ref	-
≥4	87 (87.9)	12 (12.1)	**2.88 (1.31-6.31)**	**2.49 (1.08-5.74)**	**0.03**
Inconsistent condom use: regular male partner(s)	103 (83.7)	20 (16.3)	1.18 (0.73-1.91)	-	-
Inconsistent condom use: casual partner(s)	77 (78.6)	21 (21.4)	0.89 (0.53-1.49)	-	-
HIV knowledge composite score, per 20% increase	85 (15)	85 (23)	1.38 (0.85-2.24)	-	-
Social support for condom use, composite score, per 20% increase	63 (50)	63 (63)	0.97 (0.75-1.25)	-	-

[1]vs. REF Muslim, other, or no religion.
[2]vs. REF: Bisexual, MSM, straight, other.
[3]vs. REF: Insertive, versatile or other.
[4]vs. REF: Married or other.
[5]vs. REF: Had male partners only in the past 12 months.
All variables listed in this table were considered for inclusion into the multivariate models. The same variables were considered for inclusion in Douala and Yaoundé multivariate models.
Final model did not include condom use variables since they substantially reduced sample size. The HIV knowledge composite (%) was constructed by taking the sum of the number of correct responses to 13 items and converting it to a percentage. The social support for condom use composite (%) was created by dichotomizing each of the 8 items into yes (strongly agree and agree) and no (disagree and strongly disagree) then taking the sum and converting it into a percentage.
aOR, adjusted odds ratio; CBO, community-based organization; CI, confidence interval; HIV, Human Immunodeficiency Virus; IQR, interquartile range; MSM, men who have sex with men; OR, odds ratio; RDS, respondent-driven sampling; STI, sexually transmitted infection.

After RDS-weighting, having higher than secondary education (aOR: 3.51, 95% CI: 1.32-9.34), ever accessing a community-based HIV service for MSM (aOR: 3.37, 95% CI: 1.57-7.24) and having four or more male (oral or anal) sexual partners in the past 12 months compared to one to three male sexual partners (aOR: 2.49, 1.08-5.74) were associated with ever testing for HIV after adjusting for age and sexual role preference. Sensitivity analysis demonstrated that clustering by seed did not affect the statistical significance of any of the associations in the multivariate model.

Ever accessing a community-based HIV service for MSM and having four or more male sexual partners in the past 12 months were also positively and independently associated with HIV testing in the past 12 months in a separate multivariate RDS-weighted Douala model (results not presented).

Factors associated with prior HIV testing: Yaoundé

The bivariate and multivariate associations with ever testing for HIV in Yaoundé are presented in Table 4. Consistent with Douala, bivariate RDS-weighted analysis showed that 1.24 times higher odds of ever testing for HIV per year increase in age for men aged 18 to 29 (OR: 1.24; 95% CI: 1.09-1.40) then declined per year increase in age from age 30 (OR: 0.73, 95% CI: 0.59-0.90). Having higher than secondary education (OR: 9.54, 95% CI: 1.75-52.13) and more HIV-related knowledge (OR: 2.13, 95% CI: 1.17-3.87) were also strongly associated with ever being tested for HIV. Receptive sexual role preference (OR: 0.45, 95% CI: 0.23-0.89) and ever receiving free condoms (OR: 2.19, 95% CI: 1.08-4.43) were only associated with ever testing for HIV in the RDS-naïve bivariate model. Inconsistent condom use with casual partners was associated with reduced likelihood of ever testing for HIV only in the RDS-naïve model (OR: 0.33, 95% CI: 0.14-0.76). Unlike findings from Douala, having accessed a CBO HIV service for MSM was not significantly correlated with HIV testing in Yaoundé bivariate models ($p > 0.05$).

In multivariate RDS-weighted analysis, age was positively associated with ever tested for HIV in Yaoundé for men aged 18 to 29 (aOR: 1.16, 95% CI: 1.03-1.32; aOR: 0.81, 95% CI: 0.64-1.03 if aged ≥30) as was having higher than secondary education (aOR: 7.96, 95% CI: 1.31-48.41). Ever receiving free condoms was independently associated with HIV testing in the RDS-naïve multivariate model (aOR: 2.31, 95% CI: 1.06-5.02) but not the RDS-adjusted multivariate model. Sensitivity analysis demonstrated that clustering by seed did not affect the statistical significance of any of the associations in the multivariate model.

In a separate RDS-weighted multivariate Yaoundé model, having higher than secondary education was the only variable significantly associated with being tested for HIV in the past 12 months (results not presented).

Discussion

Overall, HIV testing uptake was encouragingly high among MSM in our study compared to studies in other sub-Saharan African settings [7,8,12,13,15,16]. However, a substantial proportion of men had not been tested at least annually, and a significant proportion of men who tested HIV seropositive had never had an HIV test (6.9% in Douala, 17.4% in Yaoundé). Rapid transmission within sexual networks can occur during acute infection, and high HIV prevalence and risk behaviors has been observed among MSM in Douala and Yaoundé [5]. Given these findings, increased programmatic and research efforts will be required to encourage regular HIV testing among all MSM and link diagnosed MSM to integrated care services. Targeting sub-populations less likely to be tested such as men who have lower socioeconomic status or who are younger, and encouraging more frequent testing during times of higher HIV risk, such as testing every 3 to 6 months when men are in non-monogamous sexual relationships, may also increase engagement in the HIV treatment cascade [38].

Testing in the past 12 months and lifetime frequency of HIV testing was lower among MSM in Yaoundé compared to Douala. It was also observed in Yaoundé that inconsistent condom use with casual partners was associated with never having been tested for HIV. The majority of men in Yaoundé last accessed HIV testing at a public or private hospital, which is unsurprising given that the single community-based testing centre (CAMNAFAW) in Yaoundé was only open for a few months prior to this study [EP personal communication]. In contrast, participants in Douala who had accessed the local CBO Alternatives-Cameroun, which has been providing tailored HIV services for MSM since 2006 [23], were more likely to have been tested for HIV in the past 12 months and to have been last tested at this CBO. Additionally, more men reported being willing to return to this CBO for future testing compared to men last tested at a hospital or other testing sites, though future studies powered to assess differences in willingness to return to HCT sites by type of site are required.

A finding specific to Douala was that men with one to three male partners in the past 12 months were less likely to ever get tested than men with four or more male partners, which may reflect a personal assessment of lower risk of HIV acquisition. Inconsistent use of condoms and CCL are common among MSM in Douala [5], and network factors such as community viral load, concurrent relationships, and delayed linkage to care may propagate rapid HIV transmission even among men with two to three sexual partners [1,39]. This further highlights the importance of regular HIV testing for all MSM who are not in long-term monogamous partnerships during counseling, outreach and media campaigns [16,19,40,41].

Table 4 Bivariate and multivariate models of the factors associated with HIV testing among men who have sex with men (MSM) in Yaoundé (n = 238), Cameroon 2011

	Ever tested for HIV n (%)	Never tested for HIV n (%)	RDS-weighted OR (95% CI)	RDS-weighted OR (95% CI)	p-value
Total	197 (82.8)	41 (17.2)			
Age (years)					
18–29 (per year increase in age)	158 (81.0)	37 (19.0)	**1.24 (1.09-1.40)**	**1.16 (1.03-1.32)**	**0.01**
≥30 (per year increase in age)	39 (90.7)	4 (9.3)	**0.73 (0.59-0.90)**	0.81 (0.64-1.03)	0.09
Highest level of education attained					
≤Secondary	124 (76.1)	39 (23.9)	Ref	Ref	-
University or technical studies	73 (97.3)	2 (2.7)	**9.54 (1.75-52.13)**	**7.96 (1.31-48.41)**	**0.03**
Occupational status					
Student or apprentice	71 (80.7)	17 (19.3)	Ref	-	-
Employed	104 (86.0)	17 (14.1)	1.43 (0.63-3.28)	-	-
Unemployed	22 (75.9)	7 (24.1)	0.71 (0.23-2.20)	-	-
Christian religion[1]	185 (84.1)	35 (15.9)	1.60 (0.49-5.25)	-	-
Sexual orientation: Gay[2]	63 (86.3)	10 (13.7)	1.05 (0.44-2.49)	-	-
Sexual role preference: Receptive[3]	55 (74.3)	19 (25.7)	0.50 (0.23-1.09)	0.53 (0.21-1.30)	0.2
Relationship status: Single[4]	157 (80.9)	37 (19.1)	0.55 (0.15-2.04)	-	-
Ever accessed CBO HIV service for MSM	88 (88.0)	12 (12.0)	1.52 (0.66-3.48)	-	-
Ever received free condoms	138 (86.8)	21 (13.2)	1.69 (0.77-3.75)	1.61 (0.66-3.94)	0.3
In the last 12 months:					
Any STI symptom	81 (85.3)	14 (14.7)	1.37 (0.62-3.04)	-	-
Had male and female sexual partners[5]	91 (82.7)	19 (17.3)	0.64 (0.34-1.20)	-	-
Number of male partners					
1-3	106 (80.3)	26 (19.7)	Ref	-	-
≥4	91 (85.9)	15 (14.2)	1.51 (0.69-3.31)	-	-
Inconsistent condom use: regular male partner(s)	125 (83.3)	25 (16.7)	0.95 (0.56-1.59)	-	-
Inconsistent condom use: casual partner(s)	73 (76.0)	23 (24.0)	0.69 (0.45-1.07)	-	-
HIV knowledge composite score, per 20% increase	85 (15)	77 (15)	**2.13 (1.17-3.87)**	-	-
Social support for condom use, composite score, per 20% increase	81 (50)	63 (38)	0.86 (0.62-1.21)	-	-

[1]vs. REF Muslim, other, or no religion.
[2]vs. REF: Bisexual, MSM, straight, other.
[3]vs. REF: Insertive, versatile or other.
[4]vs. REF: Married or other.
[5]vs. REF: Had male partners only in the past 12 months.
All variables listed in this table were considered for inclusion into the multivariate models. The same variables were considered for inclusion in Douala and Yaoundé multivariate models.
Final model did not include condom use variables since they substantially reduced sample size. The HIV knowledge composite (%) was constructed by taking the sum of the number of correct responses to 13 items and converting it to a percentage. The social support for condom use composite (%) was created by dichotomizing each of the 8 items into yes (strongly agree and agree) and no (disagree and strongly disagree) then taking the sum and converting it into a percentage.
aOR, adjusted odds ratio; CBO, community-based organization; CI, confidence interval; HIV, Human Immunodeficiency Virus; IQR, interquartile range; MSM, men who have sex with men; OR, odds ratio; RDS, respondent-driven sampling; STI, sexually transmitted infection.

In order to improve the delivery and uptake of HIV testing moving forward, identifying geographic areas in which confidential MSM-sensitive HIV testing is not available, and expanding access to affordable, confidential and MSM-friendly HCT sites, which provide linkage to affordable and appropriate HIV care, treatment and related services, may be useful in reducing the number of undiagnosed MSM and in disrupting HIV transmission among MSM in Cameroon [6,23,42]. Evaluating the feasibility of couples-based testing, partner-notification services, and rapid testing may also facilitate HIV testing uptake among MSM [43]. Community leaders and the media were successfully engaged in Senegal during early planning stages for responding to the HIV epidemic among MSM, and lessons learned from that context may improve community engagement in Cameroon [44].

Approximately half of our participants were last tested in mainstream hospitals or clinics, however only 6.5% of participants received any information on HIV testing from a doctor or nurse prior to their last test. Strategies to increase provider-initiated HIV testing and sensitivity training for healthcare workers regarding MSM-specific health needs may be useful [45,46]. Stigma and discrimination towards MSM due to sexual orientation and concerns of confidentiality in mainstream healthcare settings have been documented in Cameroon [23,25] and may deter some men from accessing these services. MSM in Douala have reported distrust of health professionals, and difficulty knowing if a doctor is “gay-friendly” [23]. In a study conducted in southern Africa, few (17%) MSM reported ever disclosing same-sex practices to a health professional [26]. As in other settings, the criminalization of male-to-male sexual practices may pose a policy barrier to providing HIV prevention and treatment services for MSM [23,27,32,47]. Protecting the dignity and rights of MSM, decreasing marginalization, and allowing a safe environment for individuals to disclose their sexual orientation and HIV status, are likely to increase access to HIV prevention, treatment and care [48]. Decentralized testing programs such as home-based self-testing may also be necessary in increasing testing coverage and testing frequency especially among MSM who prefer anonymity and non-disclosure of their sexual practices with a healthcare provider [49].

Social vulnerability as measured by lower educational level was strongly associated with never having been tested and not having been tested in the past 12 months in both cities after adjusting for age in multivariate analysis, which is consistent with previous studies [8,21,50]. Higher education may provide higher income, better health literacy and better access to healthcare services, such as access to HIV testing programs. In future programs, targeting men with lower socioeconomic status may be a useful strategy to increase HIV testing uptake in Cameroon.

In both cities, young MSM aged 18 to 21 were least likely to report having ever been tested for HIV and HIV testing in the past 12 months compared to older men. Given these data, and that men in this age group are being diagnosed with HIV and the median age of sexual debut with another man is 19 years in our sample (IQR 17–22) [5], including young MSM in future HIV testing initiatives and HIV surveillance studies may improve testing outcomes. While a trend towards negative association between older age and ever testing was observed among men aged 30 or greater in both cities, additional research will be needed to corroborate these data due to small sample sizes in this age category.

Encouragingly, overall knowledge of HIV acquisition and transmission risks was fairly high in both cities and was not independently associated with HIV testing. Other studies have highlighted that individual HIV knowledge items may be worthy of exploration in future analyses, such as knowledge that unprotected penile-anal intercourse having higher risk of HIV transmission than penile-vaginal intercourse, and the importance of using CCLs during anal intercourse [7,10].

This study has a number of limitations. The cross-sectional design of this study does not allow us to assume causality of the associations in this study. Data on whether individuals already knew that they were living with HIV were not collected in this study; excluding men who have previously been diagnosed with HIV from the analysis may change the study results. Assessing regular testing such as testing every 12 months is an important outcome for the design and implementation of HIV testing programs; future research in Cameroon may investigate the correlates of testing for HIV in the last 12 months after excluding men already diagnosed with HIV. Given that six recruitment waves were not reached in every recruitment chain, MSM who are less likely to be socially connected to recruits of seeds, such as men who do not attend MSM CBOs, may be underrepresented in this study. Moreover, the generalizability of the findings for men living in smaller urban centers and rural settings in Cameroon is unknown given that data collection occurred in two large cities. The assumptions of RDS for the MSM population in Cameroon have not been tested in the current study or other studies in this setting to date. The modest sample size may have resulted in associations that occurred due to chance. Survey data may be subject to recall bias and social desirability bias. Despite these limitations, the data presented can contribute to the dialogue between researchers, policymakers and program implementers addressing the HIV epidemic among MSM in the region.

In order to elucidate the barriers to HIV testing, future studies may explore other unmeasured individual, social and structural facilitators and barriers to HIV testing in Cameroon such as knowledge of partner’s HIV status, attitudes towards HIV, geospatial dynamics, fear of seeking health services and the role of economic constraints [8,26]. Whether MSM are encouraged or discouraged by their social environment to get tested will require further elucidation [17,51].

Conclusions

Access to HIV testing is a key first step in successfully linking MSM to the continuum of HIV care and treatment [52,53]. These data demonstrate that subgroups of MSM such as young MSM and men with less education may need to be targeted when designing future HIV prevention programs and research studies and that innovative approaches to reaching this marginalized population may be useful. Given the legal context, increasing appropriate

periodicity of HIV testing among MSM in Cameroon necessitates continued investment in decentralized, culturally and clinically competent, and confidential health services. This may include the expansion of MSM-friendly CBOs providing HCT services to provide more complex HIV service delivery or strengthening the CBOs' referral system into care and treatment services. In addition, reducing structural barriers such as homophobia may help to optimize the response against the HIV epidemic in the region. Ultimately, given the levels of HIV testing and high HIV burden in Cameroon, optimizing the safe and effective provision and uptake of ART-based prevention and treatment approaches through improved uptake of HIV testing is paramount in changing the trajectory of the HIV epidemic among these men and within their sexual networks.

Competing interests
The authors declare that they have no competing interests.

Authors' contributions
SCB, SK, LM, and IM conceived and designed the study with input from YY, JE and VM. LM, SK, IM, YY and JE led the implementation. ML supervised sample collection and immunoassays. JP, EP, AG and SB developed the analytic plan. JP performed the statistical analysis with support from SCK and wrote the first draft of the manuscript. All authors collaborated in writing sections of the manuscript, gave critical input and assisted with data analysis. All authors read and approved the final manuscript.

Acknowledgements
We gratefully acknowledge the study participants and the study staff. This study was implemented by the National HIV/AIDS Prevention Programme (HAPP), a collaborative initiative involving the Association Camerounaise pour le Marketing Social (ACMS), CARE International-Cameroon, the Cameroon Ministry of Public Health, Global Viral Forecasting Initiative (GVFI), and three community-based organizations that provide HIV prevention and health services to MSM: Alternatives-Cameroun, Humanity First and the Cameroon National Association for Family Welfare (CAMNAFAW). This study was supported by a grant from the United States Agency for International Development (USAID). Publication of this article was funded in part by the Open Access Promotion Fund of the Johns Hopkins University Libraries.
A preliminary analysis of data reported here was presented at the 7th International AIDS Society Conference, which was held from 30 June to 3 July 2013 in Kuala Lumpur, Malaysia. The abstract was entitled "Optimizing multicomponent interventions for men who have sex with men (MSM) in Cameroon: factors associated with HIV testing".

Author details
[1]Center for Public Health and Human Rights, Department of Epidemiology, Johns Hopkins Bloomberg School of Public Health, Baltimore, USA. [2]Comité national de lutte contre le sida (CNLS), Ministère de la Sante Publique (MINSANTE), Yaoundé, Cameroon. [3]West and Central Africa region, Population Services International, Cotonou, Bénin. [4]Association Camerounaise pour le Marketing Social (ACMS), Yaoundé, Cameroon. [5]CARE International Cameroon, Yaoundé, Cameroon. [6]Alternatives-Cameroun, Douala, Cameroon. [7]Humanity First, Yaoundé, Cameroon. [8]Mosaic/Global Viral Cameroon, Yaoundé, Cameroon.

Received: 12 June 2014 Accepted: 10 November 2014
Published: 25 November 2014

References
1. Beyrer C, Baral SD, van Griensven F, Goodreau SM, Chariyalertsak S, Wirtz AL, Brookmeyer R: **Global epidemiology of HIV infection in men who have sex with men.** *Lancet* 2012, **380:**367–377.
2. Baral S, Sifakis F, Cleghorn F, Beyrer C: **Elevated risk for HIV infection among men who have sex with men in low- and middle-income countries 2000–2006: a systematic review.** *PLoS Med* 2007, **4:**e339.
3. van Griensven F, de Lind Van Wijngaarden JW, Baral S, Grulich A: **The global epidemic of HIV infection among men who have sex with men.** *Curr Opin HIV AIDS* 2009, **4:**300–307.
4. Papworth E, Ceesay N, An L, Thiam-Niangoin M, Ky-Zerbo O, Holland C, Drame FM, Grosso A, Diouf D, Baral SD: **Epidemiology of HIV among female sex workers, their clients, men who have sex with men and people who inject drugs in West and Central Africa.** *J Int AIDS Soc* 2013, **16:**18751.
5. Park JN, Papworth E, Kassegne S, Moukam L, Billong SC, Macauley I, Yomb YR, Nkoume N, Mondoleba V, Eloundou J, LeBreton M, Tamoufe U, Grosso A, Baral SD: **HIV prevalence and factors associated with HIV infection among men who have sex with men in Cameroon.** *J Int AIDS Soc* 2013, **16:**18752.
6. Marks G, Crepaz N, Senterfitt JW, Janssen RS: **Meta-analysis of high-risk sexual behavior in persons aware and unaware they are infected with HIV in the United States: implications for HIV prevention programs.** *J Acquir Immune Defic Syndr* 2005, **39:**446–453.
7. Baral S, Trapence G, Motimedi F, Umar E, Iipinge S, Dausab F, Beyrer C: **HIV prevalence, risks for HIV infection, and human rights among men who have sex with men (MSM) in Malawi, Namibia, and Botswana.** *PLoS One* 2009, **4:**e4997.
8. Nel JA, Yi H, Sandfort TG, Rich E: **HIV-untested men who have sex with men in South Africa: the perception of not being at risk and fear of being tested.** *AIDS Behav* 2013, **17 Suppl 1:**S51–S59.
9. Lane T, Raymond HF, Dladla S, Rasethe J, Struthers H, McFarland W, McIntyre J: **High HIV prevalence among men who have sex with men in Soweto, South Africa: results from the Soweto Men's Study.** *AIDS Behav* 2011, **15:**626–634.
10. Baral SD, Ketende S, Mnisi Z, Mabuza X, Grosso A, Sithole B, Maziya S, Kerrigan DL, Green JL, Kennedy CE, Adams D: **A cross-sectional assessment of the burden of HIV and associated individual- and structural-level characteristics among men who have sex with men in Swaziland.** *J Int AIDS Soc* 2013, **16:**18768.
11. Batist E, Brown B, Scheibe A, Baral SD, Bekker LG: **Outcomes of a community-based HIV-prevention pilot programme for township men who have sex with men in Cape Town, South Africa.** *J Int AIDS Soc* 2013, **16:**18754.
12. Baral S, Adams D, Lebona J, Kaibe B, Letsie P, Tshehlo R, Wirtz A, Beyrer C: **A cross-sectional assessment of population demographics. HIV risks and human rights contexts among men who have sex with men in Lesotho.** *J Int AIDS Soc* 2011, **14:**36.
13. Wade AS, Kane CT, Diallo PA, Diop AK, Gueye K, Mboup S, Ndoye I, Lagarde E: **HIV infection and sexually transmitted infections among men who have sex with men in Senegal.** *AIDS* 2005, **19:**2133–2140.
14. Dramé FM, Crawford EE, Diouf D, Beyrer C, Baral SD: **A pilot cohort study to assess the feasibility of HIV prevention science research among men who have sex with men in Dakar, Senegal.** *J Int AIDS Soc* 2013, **16**(Suppl 3):18753.
15. Vu L, Andrinopoulos K, Tun W, Adebajo S: **High levels of unprotected anal intercourse and never testing for HIV among men who have sex with men in Nigeria: evidence from a cross-sectional survey for the need for innovative approaches to HIV prevention.** *Sex Transm Infect* 2013, **89:**659–665.
16. Merrigan M, Azeez A, Afolabi B, Chabikuli ON, Onyekwena O, Eluwa G, Aiyenigba B, Kawu I, Ogungbemi K, Hamelmann C: **HIV prevalence and risk behaviours among men having sex with men in Nigeria.** *Sex Transm Infect* 2011, **87:**65–70.
17. Lorente N, Henry E, Fugon L, Yomb Y, Carrieri MP, Eboko F, Spire B: **Proximity to HIV is associated with a high rate of HIV testing among men who have sex with men living in Douala, Cameroon.** *AIDS Care* 2012, **24:**1020–1027.
18. Finlayson TJ, Le B, Smith A, Bowles K, Cribbin M, Miles I, Oster AM, Martin T, Edwards A, Dinenno E: **HIV risk, prevention, and testing behaviors among men who have sex with men–National HIV Behavioral Surveillance System, 21 U.S. cities, United States, 2008.** *MMWR Surveill Summ* 2011, **60:**1–34.
19. Beyrer C, Trapence G, Motimedi F, Umar E, Iipinge S, Dausab F, Baral S: **Bisexual concurrency, bisexual partnerships, and HIV among Southern African men who have sex with men.** *Sex Transm Infect* 2010, **86:**323–327.

20. Sandfort TG, Nel J, Rich E, Reddy V, Yi H: **HIV testing and self-reported HIV status in South African men who have sex with men: results from a community-based survey.** *Sex Transm Infect* 2008, **84:**425–429.
21. Knox J, Sandfort T, Yi H, Reddy V, Maimane S: **Social vulnerability and HIV testing among South African men who have sex with men.** *Int J STD AIDS* 2011, **22:**709–713.
22. Geibel S, Tun W, Tapsoba P, Kellerman S: **HIV vulnerability of men who have sex with men in developing countries: Horizons studies, 2001–2008.** *Public Health Rep* 2010, **125:**316–324.
23. Kalamar M, Maharaj P, Gresh A: **HIV-prevention interventions targeting men having sex with men in Africa: field experiences from Cameroon.** *Cult Health Sex* 2011, **13:**1135–1149.
24. Altman D, Aggleton P, Williams M, Kong T, Reddy V, Harrad D, Reis T, Parker R: **Men who have sex with men: stigma and discrimination.** *Lancet* 2012, **380:**439–445.
25. Human Rights Watch: **Criminalizing Identities: Rights abuses in Cameroon based on sexual Orientation and gender identity.** In *Book Criminalizing Identities: Rights abuses in Cameroon based on sexual Orientation and gender identity.* New York, NY: Human Rights Watch; 2010.
26. Fay H, Baral SD, Trapence G, Motimedi F, Umar E, Iipinge S, Dausab F, Wirtz A, Beyrer C: **Stigma, health care access, and HIV knowledge among men who have sex with men in Malawi, Namibia, and Botswana.** *AIDS Behav* 2011, **15:**1088–1097.
27. Poteat T, Diouf D, Drame FM, Ndaw M, Traore C, Dhaliwal M, Beyrer C, Baral S: **HIV risk among MSM in Senegal: a qualitative rapid assessment of the impact of enforcing laws that criminalize same sex practices.** *PLoS One* 2011, **6:**e28760.
28. Rispel LC, Metcalf CA, Cloete A, Moorman J, Reddy V: **You become afraid to tell them that you are gay: health service utilization by men who have sex with men in South African cities.** *J Public Health Policy* 2011, **32**(Suppl 1):S137–S151.
29. Spielberg F, Branson BM, Goldbaum GM, Lockhart D, Kurth A, Celum CL, Rossini A, Critchlow CW, Wood RW: **Overcoming barriers to HIV testing: preferences for new strategies among clients of a needle exchange, a sexually transmitted disease clinic, and sex venues for men who have sex with men.** *J Acquir Immune Defic Syndr* 2003, **32:**318–327.
30. Levy ME, Wilton L, Phillips G 2nd, Glick SN, Kuo I, Brewer RA, Elliott A, Watson C, Magnus M: **Understanding Structural Barriers to Accessing HIV Testing and Prevention Services Among Black Men Who Have Sex with Men (BMSM) in the United States.** *AIDS Behav* 2014, 18:972–996.
31. Yasin F, Delegchoimbol A, Jamiyanjamts N, Sovd T, Mason K, Baral S: **A Cross-Sectional Evaluation of Correlates of HIV Testing Practices Among Men Who Have Sex with Men (MSM) in Mongolia.** *AIDS Behav* 2013, **17:**1378–1385.
32. Millett GA, Jeffries WL, Peterson JL, Malebranche DJ, Lane T, Flores SA, Fenton KA, Wilson PA, Steiner R, Heilig CM: **Common roots: a contextual review of HIV epidemics in black men who have sex with men across the African diaspora.** *Lancet* 2012, **380:**411–423.
33. Beyrer C, Sullivan PS, Sanchez J, Dowdy D, Altman D, Trapence G, Collins C, Katabira E, Kazatchkine M, Sidibe M, Mayer KH: **A call to action for comprehensive HIV services for men who have sex with men.** *Lancet* 2012, **380:**424–438.
34. Baral S, Logie CH, Grosso A, Wirtz AL, Beyrer C: **Modified social ecological model: a tool to guide the assessment of the risks and risk contexts of HIV epidemics.** *BMC Public Health* 2013, **13:**482.
35. National AIDS Control Commitee: **UNGASS Coutry Progress Report: Rapport national de suivi de la declaration politique sur le VIH/SIDA Cameroun.** In *Book UNGASS Coutry Progress Report: Rapport national de suivi de la declaration politique sur le VIH/SIDA Cameroun.* Yaoundé, Cameroon: Joint United Nations Programme on HIV/AIDS; 2012.
36. Heckathorn DD: **Respondent-driven sampling: A new approach to the study of hidden populations.** *Soc Probl* 1997, **44:**174–199.
37. Volz E, Heckathorn DD: **Probability Based Estimation Theory for Respondent Driven Sampling.** *J Off Stat* 2008, **24:**79–97.
38. Centers for Disease Control and Prevention: **HIV testing among men who have sex with men–21 cities, United States, 2008.** *MMWR Morb Mortal Wkly Rep* 2011, **60:**694–699.
39. Mah TL, Halperin DT: **Concurrent sexual partnerships and the HIV epidemics in Africa: evidence to move forward.** *AIDS Behav* 2010, **14:**11–16. dicussion 34–17.
40. Sandoy IF, Dzekedzeke K, Fylkesnes K: **Prevalence and correlates of concurrent sexual partnerships in Zambia.** *AIDS Behav* 2010, **14:**59–71.
41. Das M, Chu PL, Santos GM, Scheer S, Vittinghoff E, McFarland W, Colfax GN: **Decreases in community viral load are accompanied by reductions in new HIV infections in San Francisco.** *PLoS One* 2010, **5:**e11068.
42. Mayer KH, Pape JW, Wilson P, Diallo DD, Saavedra J, Mimiaga MJ, Koenig S, Farmer P: **Multiple determinants, common vulnerabilities, and creative responses: addressing the AIDS pandemic in diverse populations globally.** *J Acquir Immune Defic Syndr* 2012, **60**(Suppl 2):S31–S34.
43. Henley C, Forgwei G, Welty T, Golden M, Adimora A, Shields R, Muffih PT: **Scale-up and case-finding effectiveness of an HIV partner services program in Cameroon: an innovative HIV prevention intervention for developing countries.** *Sex Transm Dis* 2013, **40:**909–914.
44. Beyrer C, Wirtz AL, Walker D, Johns B, Sifakis F, Baral SD: **Scenario 3 Country Studies: Kenya, Malawi, and Senegal.** In *The Global HIV Epidemics among Men who have Sex with Men.* Washington DC: The World Bank; 2011:81–110.
45. van der Elst EM, Smith AD, Gichuru E, Wahome E, Musyoki H, Muraguri N, Fegan G, Duby Z, Bekker LG, Bender B, Graham SM, Operario D, Sanders EJ: **Men who have sex with men sensitivity training reduces homoprejudice and increases knowledge among Kenyan healthcare providers in coastal Kenya.** *J Int AIDS Soc* 2013, **16:**18748.
46. van der Elst EM, Gichuru E, Omar A, Kanungi J, Duby Z, Midoun M, Shangani S, Graham SM, Smith AD, Sanders EJ, Operario D: **Experiences of Kenyan healthcare workers providing services to men who have sex with men: qualitative findings from a sensitivity training programme.** *J Int AIDS Soc* 2013, **16:**18741.
47. International Lesbian Gay Bisexual Trans and Intersex Association: *State Sponsored Homophobia report 2011: A World Survey of Laws Criminalising Same-Sex Sexual Acts between Consenting Adults.* Geneva, Switzerland: International Lesbian, Gay, Bisexual, Trans and Intersex Association; 2012.
48. Betron M, Gonzalez-Figueroa E: *Gender Identity, Violence, and HIV among MSM and TG: A Literature Review and a Call for Screening.* Washington D.C.: Futures Group International, USAID. Health Policy Initiative, Task Order 1; 2009.
49. Pant Pai N, Sharma J, Shivkumar S, Pillay S, Vadnais C, Joseph L, Dheda K, Peeling RW: **Supervised and unsupervised self-testing for HIV in high- and low-risk populations: a systematic review.** *PLoS Med* 2013, **10:**e1001414.
50. MacKellar DA, Valleroy LA, Secura GM, Bartholow BN, McFarland W, Shehan D, Ford W, LaLota M, Celentano DD, Koblin BA, Torian LV, Perdue TE, Janssen RS, Young Men's Survey Study, Group: **Repeat HIV testing, risk behaviors, and HIV seroconversion among young men who have sex with men: a call to monitor and improve the practice of prevention.** *J Acquir Immune Defic Syndr* 2002, **29:**76–85.
51. Grosso A: **Social support as a predictor of HIV testing in at-risk populations: a research note.** *J Health Hum Serv Adm* 2010, **33:**53–62.
52. Centers for Disease Control and Prevention: **Vital signs: HIV prevention through care and treatment–United States.** *MMWR Morb Mortal Wkly Rep* 2011, **60:**1618–1623.
53. Gardner EM, McLees MP, Steiner JF, Del Rio C, Burman WJ: **The spectrum of engagement in HIV care and its relevance to test-and-treat strategies for prevention of HIV infection.** *Clin Infect Dis* 2011, **52:**793–800.

doi:10.1186/1471-2458-14-1220
Cite this article as: Park *et al.*: Correlates of prior HIV testing among men who have sex with men in Cameroon: a cross-sectional analysis. *BMC Public Health* 2014 **14**:1220.

Submit your next manuscript to BioMed Central and take full advantage of:

- **Convenient online submission**
- **Thorough peer review**
- **No space constraints or color figure charges**
- **Immediate publication on acceptance**
- **Inclusion in PubMed, CAS, Scopus and Google Scholar**
- **Research which is freely available for redistribution**

Submit your manuscript at
www.biomedcentral.com/submit

BioMed Central
